# Inter-species functional compatibility of the *Theobroma cacao* and Arabidopsis FT orthologs: 90 million years of functional conservation of meristem identity genes

**DOI:** 10.1186/s12870-021-02982-y

**Published:** 2021-05-14

**Authors:** S. F. Prewitt, A. Shalit-Kaneh, S. N. Maximova, M. J. Guiltinan

**Affiliations:** 1 Department of Plant Sciences, The Pennsylvania State University, University Park, PA USA; 2 Huck Institutes of the Life Sciences, The Pennsylvania State University, University Park, PA USA

**Keywords:** *FLOWERING LOCUS T* (*FT*), Florigen, Accelerated flowering, *Theobroma cacao*

## Abstract

**Background:**

In angiosperms the transition to flowering is controlled by a complex set of interacting networks integrating a range of developmental, physiological, and environmental factors optimizing transition time for maximal reproductive efficiency. The molecular mechanisms comprising these networks have been partially characterized and include both transcriptional and post-transcriptional regulatory pathways. Florigen, encoded by *FLOWERING LOCUS T* (*FT*) orthologs, is a conserved central integrator of several flowering time regulatory pathways. To characterize the molecular mechanisms involved in controlling cacao flowering time, we have characterized a cacao candidate florigen gene, *TcFLOWERING LOCUS T* (*TcFT*). Understanding how this conserved flowering time regulator affects cacao plant’s transition to flowering could lead to strategies to accelerate cacao breeding.

**Results:**

BLAST searches of cacao genome reference assemblies identified seven candidate members of the *CENTRORADIALIS/TERMINAL FLOWER1/SELF PRUNING* gene family including a single florigen candidate. cDNA encoding the predicted cacao florigen was cloned and functionally tested by transgenic genetic complementation in the Arabidopsis *ft-10* mutant. Transgenic expression of the candidate *TcFT* cDNA in late flowering Arabidopsis *ft-10* partially rescues the mutant to wild-type flowering time. Gene expression studies reveal that *TcFT* is spatially and temporally expressed in a manner similar to that found in Arabidopsis, specifically, *TcFT* mRNA is shown to be both developmentally and diurnally regulated in leaves and is most abundant in floral tissues. Finally, to test interspecies compatibility of florigens, we transformed cacao tissues with *AtFT* resulting in the remarkable formation of flowers in tissue culture. The morphology of these in vitro flowers is normal, and they produce pollen that germinates in vitro with high rates.

**Conclusion:**

We have identified the cacao *CETS* gene family, central to developmental regulation in angiosperms. The role of the cacao’s single *FT-*like gene (*TcFT*) as a general regulator of determinate growth in cacao was demonstrated by functional complementation of Arabidopsis *ft-10* late-flowering mutant and through gene expression analysis. In addition, overexpression of *AtFT* in cacao resulted in precocious flowering in cacao tissue culture demonstrating the highly conserved function of *FT* and the mechanisms controlling flowering in cacao.

**Supplementary Information:**

The online version contains supplementary material available at 10.1186/s12870-021-02982-y.

## Background

*Theobroma cacao* is a cash crop and the sole source of cacao beans from which the primary ingredients in chocolate products, cocoa powder and cocoa butter, are derived. Its unique and critical role in the chocolate manufacturing industry makes it an important export for developing countries in Africa, Central and South Americas and in South Asia, where cacao is predominantly cultivated. Cultivation of cacao is limited by many factors including several fungal, oomycete and viral diseases that cause global losses of 20–30% [1]. Massive pathogenic losses make research and breeding for improved disease resistance crucial for the future sustainability of the crop and to improve farmer livelihoods [2]. In addition to improved disease resistance traits, cacao breeders actively pursue avenues for the improvement of cocoa quality traits such as flavor, health beneficial metabolites, climate resiliency and improved yield. However, progress in breeding programs is severely limited by cacao’s juvenile longevity and high costs of breeding typical of tree crop systems and thus the control of flowering time is of scientific and practical interest.

Native to tropical Mesoamerica [3], cacao is an understory tree principally grown in rainforest areas within 20° latitude of the equator around the world. Cacao, similar to most trees, has three primary growth phases with respect to reproductive development: Phase 1. The juvenile phase of cacao tree growth is upright and orthotropic with all aerial organs having radially phyllotaxy arising from the shoot apical meristem. The initial orthotropic growth defines the main trunk of the future tree [4]. Phase 2. After approximately 2 years, phase change occurs during which the plant transitions to the adult phase [5]. The shoot apex is consumed, and in its place arise 3–5 plagiotropic (lateral) shoot meristems [4] that give rise to branches with alternate phyllotaxy (jorquetting). Plagiotropic branches of the jorquetted tree comprise the crown of an adult cacao tree. Jorquetted cacao trees are believed to have reached competency for reproduction. Phase 3. Shortly after jorquetting, cacao transitions to reproductive Developmen*t. cacao* is cauliflorous with flowers borne from the trunk and main branches initiated from dormant axillary meristems in the axils of abscised leaves. Morphological and anatomical studies of cacao floral development have demonstrated that it shares highly conserved regulatory pathways and genes with the model plant Arabidopsis [6]. This study extends the knowledge of the mechanisms controlling the transition of cacao meristems from vegetative to floral by characterizing the function of genes encoding key regulatory proteins involved in phase-change dependent floral induction.

The transition of meristems from vegetative to floral development is controlled by the coincidence of developmental, physiological, and environmental stimuli cascading through a complex set of interacting networks integrating these signals. Initial studies into the mechanisms of floral transition demonstrated the existence of a conserved mobile signal, florigen, produced in leaves and transmitted to shoot meristems in response to photoperiod [7–9]. Florigen became the long-sought ‘holy grail’ of plant physiology until the current century when Eliezer Lifschitz and co-authors demonstrated a 1:1 genetic relationship between florigen and tomato *FLOWERING LOCUS T (FT)* ortholog, *SINGLE FLOWER TRUSS* (*SFT*) [10]. In an impressive set of experiments the authors demonstrated *SFT* produces a graft-transmissible stimulus that promotes flowering in addition to other pleotropic effects in both photoperiodic and day-neutral species thereby substituting for a diverse set of environmental stimuli. Importantly, the authors could detect SFT protein but not transgenic *SFT* mRNA in receptor tissues. Following studies demonstrated the vascular movement of FT ortholog proteins from synthetic leaf tissue to functional apical tissue (flowering) in model plant Arabidopsis (AtFT) [11–13] and in rice [14]. This demonstrated that FT orthologs are florigens, conserved mobile signals regulating flowering time in response to photoperiod in flowering plants.

*FLOWERING LOCUS T* (*FT*) is a member of the *CENTRORADIALIS/TERMINAL FLOWER1/SELF PRUNING* (*CETS*) gene family in plants [10]. In addition to its florigenic role in the photoperiodic control of flowering time, *FT* is an important integrator of several pathways known to cause the transition to reproductive growth including the ambient temperature, autonomous and vernalization pathways [15]. *FT* has also been shown to have pleiotropic activity and was recently defined as a general growth regulator that harmonizes plant developmental processes [10, 16].

Extensive studies confirming *FT’s* control of flowering time have led to biotechnological and agronomic approaches to accelerate and control flower development and fruit set [17]. For example, ectopic overexpression of the *FT* gene in transgenic long-generation plants has been used to accelerate flowering to shorten generation times to aid breeding programs. Strategies including overexpression, inducible expression and virus-based expression of *FT* have been shown to promote early flowering in several species including trees such as poplar, cotton, and apple [18–23].

Here, we describe our work to identify cacao’s *CETS* gene candidates and characterize cacao’s candidate *FT* gene (Tc05v2_g009810). We demonstrate that cacao’s candidate *TcFT* can partially rescue the late-flowering phenotype in the Arabidopsis *ft-10* mutant. Gene expression analysis suggests that *TcFT*’s leaf expression is both developmentally and diurnally regulated in a manner similar to the expression of florigenic orthologs in several species. In our analysis, we also find that similar to expression in Arabidopsis, *TcFT* mRNA is most abundant in tissues formed post-transition to flowering suggesting that *TcFT* stabilizes reproductive development in cacao. Finally, cacao somatic embryos stably expressing *AtFT* were able to develop flowers in in vitro culture. Together our results provide evidence that the major mechanisms regulating flowering are highly conserved and inter-compatible between the model plant Arabidopsis and cacao, species estimated to have diverged approx. 90 million years ago [24, 25].

## Results

### Identifying the cacao *CETS* gene family

Using Arabidopsis FT, TFL1 and CENTRORADIALIS (ATC) protein sequences as queries, we identified seven cacao *CETS* genes with e-values less than 5 × 10^− 16^ in BLASTp searches in *Theobroma cacao* Belizian Criollo B97–61/B2 v2 (Criollo) genome ([26, 27]; Table S1), and six putative *CETS* genes in Matina1–6 genome ([28, 29]; Table S2).

**Fig. 1:**
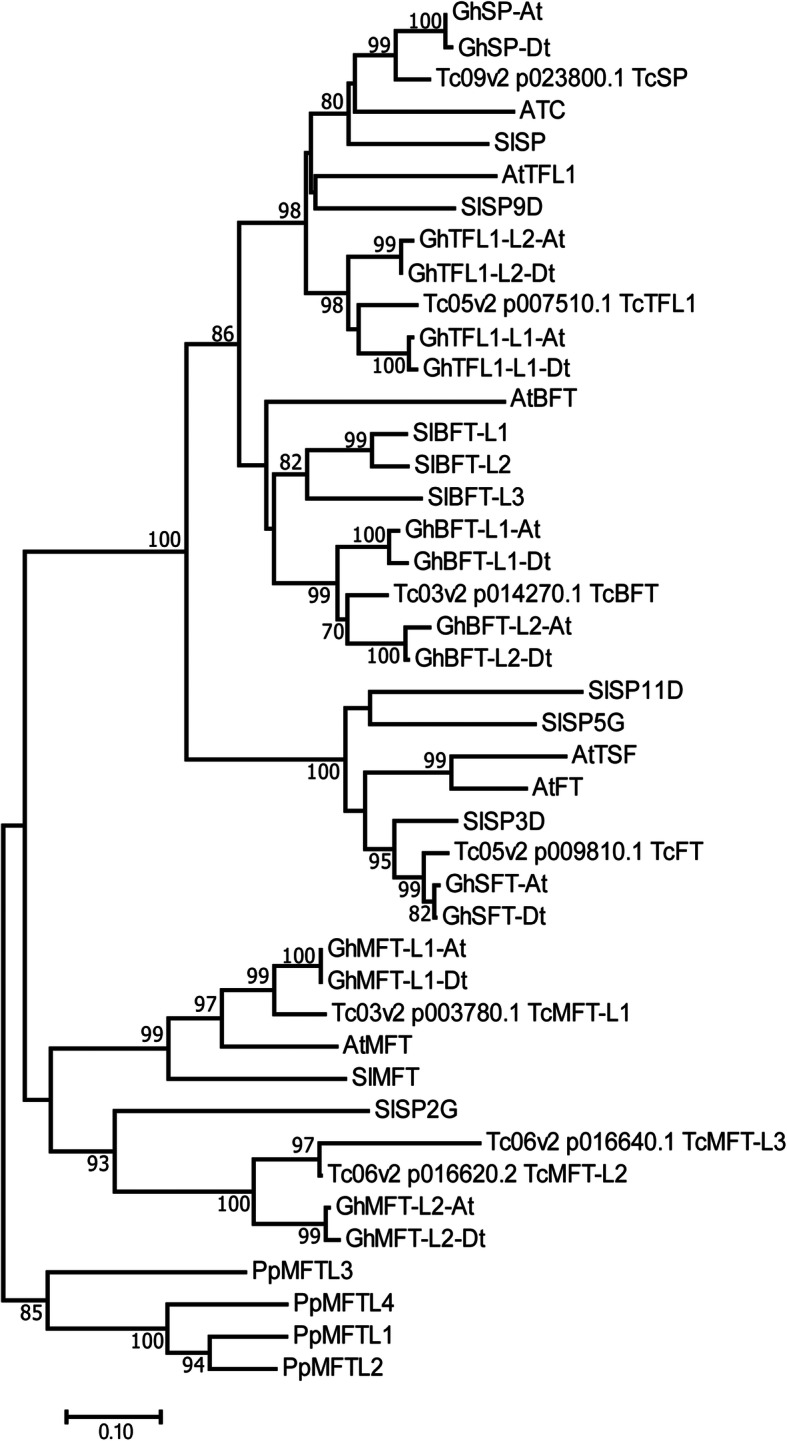
*T. cacao* CETS are organized into three major clades. Protein sequences of 43 CETS proteins including 4 moss (embryophyte), 10 tomato (eudicot, rosid), 6 Arabidopsis (eudicot, Brassicaceae), 16 cotton (eudicot, Brassicale-Malvales), and 7 cacao (eudicot, Brassicale-Malvales) were used to infer the evolutionary history of cacao CETS. The phylogenetic test used was the Bootstrap by N-J method. Dendrogram branches are labeled with percentage of 1000 iterations. The scale bar reflects the frequency of amino acid substitutions determined by the Poisson correction method

**Fig. 2:**
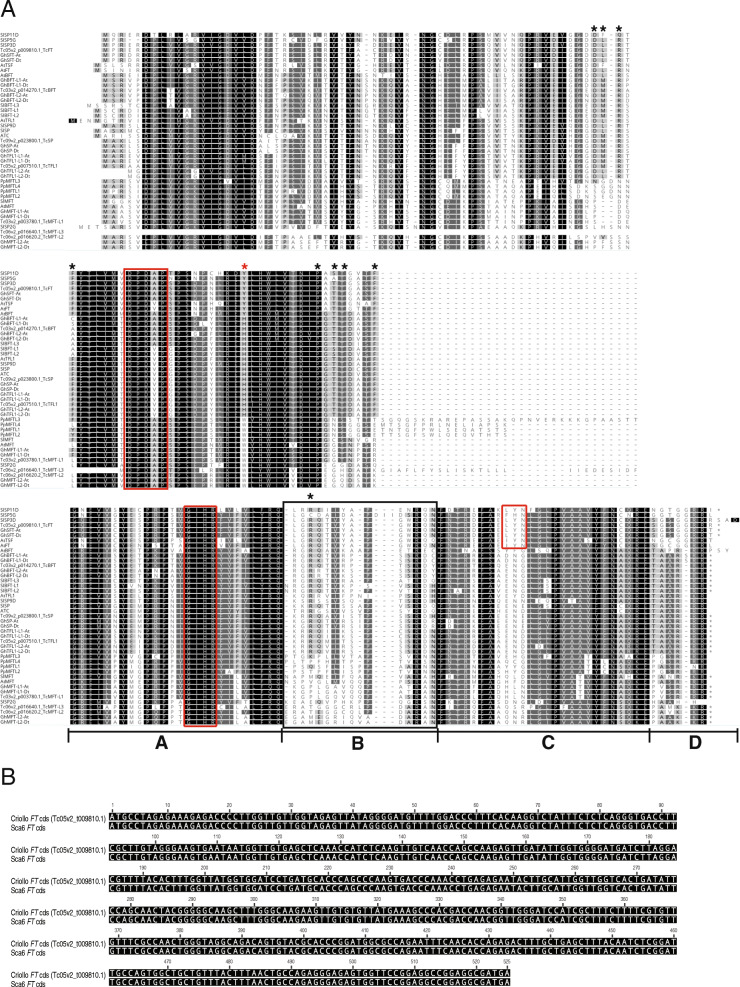
Multiple sequence alignment of CETS proteins. **a** Amino acid alignment of the CETS proteins from *Physcomiterella patens* (*Pp*), *Arabidopsis thaliana* (*At*), *Solanum lycoperscium* (*Sl*), *Gossypium hirsutum* (*Gh*), and *Theobroma cacao* (*Tc*) is displayed. The red asterisk indicates the important His-88/Tyr-85 residue critical for determining floral activating or repressive activity. The black asterisks mark residues shown to interact with 14–3-3 proteins. Red boxes highlight the conserved DPDxP, GxHR and L/IYN motifs, respectively. A black box marks the external loop portion of the ligand binding domain. Segments A-D of exon 4 as defined in (34) are underlined and labeled. Protein, species, and accession numbers for aligned sequences are listed in Supplemental Table 3 (Table S3). **b** DNA coding sequence (cds) alignment of *T. cacao* Criollo *FT* (Tc05v2_g009810, reference genome) and Scavina6 *FT* (study genotype). Scavina6 *FT* coding sequence is a consensus of alignment of cloning sequencing results (4 clones) to Criollo *FT*. Clone sequences had 100% identity to both the consensus and (as pictured) Criollo’s *FT* coding sequencing (data not shown)

The predicted full-length polypeptides of the candidate cacao CETS proteins were phylogenetically analyzed alongside CETS proteins from Arabidopsis, cotton, tomato, and moss (protein names and IDs in Table S3). Consistent with previous analyses of CETS proteins in other species, cacao CETS assort into three distinct clades: MOTHER OF FT AND TFL1- LIKE (MFT-L), FLOWERING LOCUS T-LIKE (FT-L) and TERMINAL FLOWER 1/SELF PRUNING-LIKE (TFL1/SP-L) (Fig. 1) [30–33]. The Criollo genome contains three putative CETS, Tc03v2_g003780, Tc06v2_g016620 and Tc06v2_g016640, grouped within the MFT-L subgroup of the family. A single putative protein, Tc05v2_g009810, designated TcFT, comprises the FT-L subgroup in cacao and shares 76.4% amino acid sequence identity with AtFT (Fig. 2). Three cacao CETS are grouped in the TFL1/SP-L clade. One candidate TcTFL1, Tc05v2_g007510, shares 71.1% amino acid sequence identity with AtTFL1. Tc09v2_g023800, candidate TcSP, is sub-grouped within the TFL1/SP group with SlSP and Arabidopsis ATC and shares 80% sequence identity with ATC (Fig. 1 and Table S1). Candidate TcBFT, Tc03v2_g014270, is the final TFL1/SP-L cacao CETS and resides in a subgroup of this group alongside Arabidopsis BROTHER OF FT AND TFL1 (Fig. 1).

CETS proteins contain two domains, a highly conserved anion-binding site and an external loop (exon 4 segment B), shown to be critical to function [34, 35]. Our multiple sequence alignment (Fig. 2a) demonstrates that each of the seven identified Criollo CETS predicted polypeptide sequences retain both functionally important domains and retain conservation of the conserved short DPDxP and GxHR motifs [36] within these domains. In addition, candidate TcFT (Tc05v2_g009810) is the only cacao CETS conserved in the Tyr-85 and exon 4 segments B and D defined to be essential for FT function [34].

### Expression of *TcFT* is developmentally regulated in cacao leaves

**Fig. 3:**
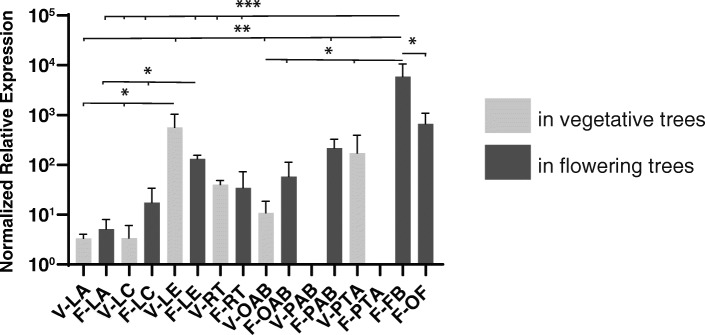
Expression of *TcFT* in various tissues of vegetative and flowering Scavina-6 trees. Bar charts illustrate the relative expression level of *TcFT* in leaves, roots, buds, apices, and floral tissues. The geometric mean of control genes *TcTUB1, TcCULLIN,* and *TcSUMO* expression was used to normalize *TcFT* expression. The log of expression values is shown and was scaled to the sample having minimum expression. V = vegetative, F = Flowering, LA = Stage A (young) leaf, LC = Stage C (intermediate) leaf, LE = Stage E (mature) leaf, RT = root, OAB = orthotropic (main trunk) axillary bud, PAB = plagiotropic (lateral crown branch) axillary bud, PTA = plagiotropic terminal apex, FB = floral bud and OF = open flower. A one-way ANOVA comparing each group mean to every other group mean was used to evaluate the datasets. A Tukey’s post-hoc test was used to correct for multiple comparisons. * = *p* < 0.05, ** = *p* < 0.01, and *** = *p* < 0.001

In order to characterize the gene expression profile of the candidate *TcFT,* we used RT-qPCR to measure transcript levels in multiple tissues of vegetative (1.5 year-old) and flowering (2.5–3.5 years-old) Scavina-6 trees including: leaves at developmental stages A, C, and E (defined in [37]), roots, orthotropic and plagiotropic axillary buds, plagiotropic shoot apices, floral buds and open flowers. Candidate *TcFT* is expressed in all six leaf tissue types assayed in both vegetative and flowering plants (Fig. 3). Expression was observed to be significantly higher in mature leaves (stage E) of both vegetative and flowering trees than in young (stage A) and developing leaves (stage C) of these trees. Specifically, in vegetative trees the expression in E leaves was 172-fold and 166-fold higher than expression in A and C leaves, respectively, while in adult trees, expression in E leaves was 25-fold and 7.5-fold higher than A and C leaves, respectively (*p* > 0.05, Fig. 3). These results suggest that cacao’s candidate *FT* gene expression levels increase with leaf age, similar to reports of tomato’s florigen [10]. These results are consistent with the hypothesis that candidate *TcFT* is cacao’s florigen ortholog.

### *TcFT* expression is highest in floral tissues

Comparison among all tissues assayed revealed floral tissues accumulated *TcFT* mRNA at the highest levels. We detected higher expression in floral tissue compared to vegetative and flowering tree apical tissue (terminal and axillary; Fig. 3b). *TcFT* was expressed in all tested bud, apex, and floral tissues except plagiotropic axillary buds of vegetative trees and plagiotropic terminal apices of flowering trees, where it was not detectable. Floral bud expression was 96-fold and 27-fold higher than orthotropic and plagiotropic axillary buds of flowering trees and 136-fold higher than plagiotropic terminal apices (*p* > 0.05), respectively, (Fig. 3). In addition, floral bud expression was observed to be 10-fold – 1500-fold higher than in any of the tested lead tissues (*p* > 0.01 or *p* > 1.001, Fig. 3). Extensive studies of *FT* in Arabidopsis and other species have revealed pleiotropic effects of *FT* expression. Notably, floral and fruit *AtFT* expression has been demonstrated to participate in stabilizing reproductive growth post-fertilization through reversion-blocking maintenance of recently developed inflorescence meristems [38]. Our results demonstrate that, similar to Arabidopsis, *TcFT* expression is higher in reproductive tissues compared with growing buds. This observation suggests that *TcFT* may also act to stabilize floral development in cacao.

### *TcFT* is diurnally regulated in mature cacao leaves

**Fig. 4:**
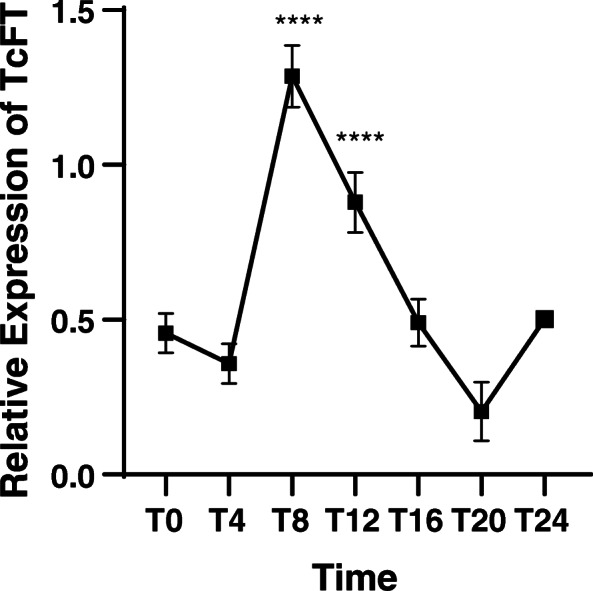
Diurnal Expression of *TcFT* in Stage E Scavina-6 cacao leaves. Column graph with connected means demonstrates the relative expression of *TcFT* in mature (Stage E) leaves measured every 4 h over a 24-h period. Expression is reported relative to *TcTUB1.* Log expression values are shown, and values are scaled relative to the sample having minimum expression. A one-way ANOVA comparing each group mean to every other group mean was used to evaluate the datasets. A Tukey’s post-hoc test was used to correct for multiple comparisons. Significant differences to T20 are shown. **** = *p* < 0.0001

In order to characterize the expression of *TcFT* in leaves in more depth, we examined its expression in fully mature (stage E), Scavina-6 leaves relative to the diurnal cycle. Stage E leaves were collected from greenhouse-grown, flowering trees every 4 h over a 24-h period. While expression of *TcFT* was generally low in these leaves, a significant spike in expression was seen 8 h post-dawn (*p* > 0.0001 to every other time point mean in one-way ANOVA) followed by a return to pre-spike expression levels throughout the remainder of the day until the next dawn. Expression at 12 h post-dawn was also significantly higher than at dawn (*p* < 0.05) and 4 h post-dawn (*p* < 0.01), but lower than at 8 h post-dawn (*p* < 0.05, Fig. 4). This result is similar to *FT* expression in several species that comprise *FT* orthologs having diurnal expression patterns. *TcFT* expression pattern peaks at midday in contrast to Arabidopsis where *FT* reaches peak expression before dusk followed by a return to baseline expression through the night [13, 39–43].

### Transgenic complementation of the Arabidopsis mutant, *ft-10* with the candidate *TcFT* gene

To determine whether candidate *TcFT* shares a highly conserved function in flowering time regulation we conducted transgenic complementation of the Arabidopsis late flowering mutant, *ft-10* (Loss-of-function of FT), which is extremely delayed in phase transition under long-day conditions. In contrast to wild-type Col-0 that flowers after development of ~ 15 leaves, *ft-10* flowering begins after > 40 rosette leaves have formed [44]. Mutant plants were transformed separately with a binary vector containing the coding sequence of the candidate *TcFT* driven by the E12-Ω modified CaMV 35S constitutive promoter [45] and with a backbone vector control (VC). Multiple independent lines of transgenic plants were identified by antibiotic resistance screening and evaluated for flowering time traits.

**Fig. 5:**
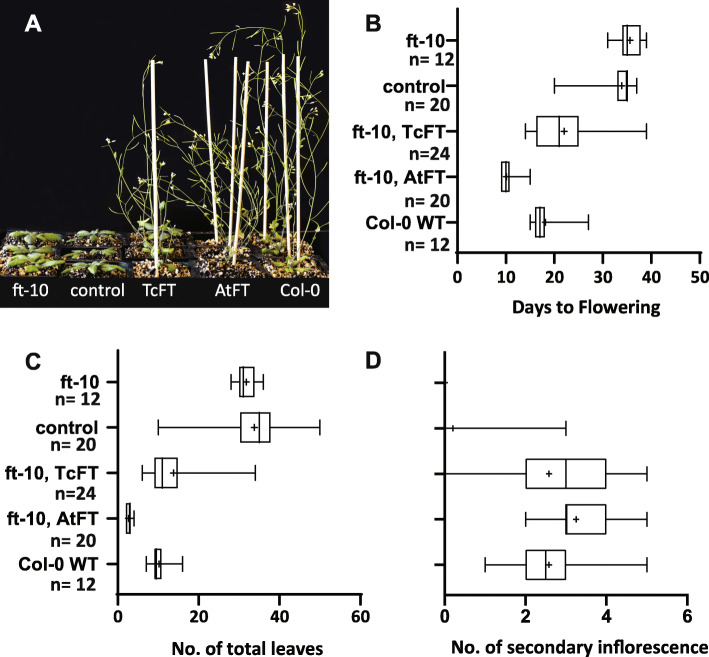
Phenotype of Arabidopsis *ft-10* mutant transformed with cacao *TcFT* gene. Images of three representative independent lines of experimental and control genotypes (**a**), from left to right: *ft-10* mutant; *ft-10*, control vector; *ft-10*, *TcFT*; *ft-10*, *AtFT*; Columbia-0 wildtype at one-month past germination. Rescue of the mutant phenotype was determined by assessing days to bolting (**b**), number of total leaves (**c**) and number of secondary inflorescences (**d**). Significant differences are shown in comparison to the control vector group and based on univariate ANOVA with Tukey’s H-S-D ad hoc analysis at the 0.05 level; ns: not significant, ****: *p* < 0.0001

Grown in 16-h day/8-h night photoperiodicity, *ft-10,* and the VC transformants flowered ~ 16 days later than wild-type Col-0 and generated 3-fold more rosette and cauline leaves and 2-times fewer secondary inflorescences in comparison to wild-type Col-0 plants (Fig. 5a-d). Arabidopsis *ft-10* mutants, expressing high levels of *TcFT,* flowered 12 to 13 days earlier than *ft-10* and T_1_ control vector plants, respectively, but 4 days later than wild-type plants (Fig. 5a and b). This is consistent with the hypothesis that *TcFT* encodes a protein that is a functional ortholog of AtFT and can interact with other proteins in Arabidopsis tissues to induce the transition from vegetative to floral development.

On average, *E-12 Ω::TcFT* transgenic plants had 13 and 15 fewer total leaves than *ft-10* and control vector plants, respectively, and 3 more leaves than wild-type (Fig. 5a and c). Expression of *E-12 Ω::TcFT* also altered the branching architecture in the *ft-10* background. While *ft-10* and control vector lines failed to produce secondary inflorescences, both *E-12 Ω::TcFT* and wild-type generated an average of 3 secondary inflorescences arising from the axillary buds of rosette leaves (Fig. 5a and d). Interestingly, independent T_1_ *E-12 Ω::AtFT* lines showed a much stronger phenotype, flowering 8 days and 8 leaves earlier than WT. These results suggest that *TcFT* is either less potent in its positive regulation of floral transition or functioned sub-optimally in the heterologous environment. We have observed this partial transgenic complementation with several other cacao genes we have functionally characterized heterologously in Arabidopsis [46–49]. Together, these data establish that *TcFT* promoted reproductive development at levels comparable to endogenous *AtFT* in WT but its overexpression in the Arabidopsis *ft-10* mutant was less potent than that of *AtFT*. Taken together, our results strongly support the conclusion that the cacao locus Tc05v2_g009810 encodes a functional ortholog of *AtFT* that exists as a single copy in the cacao genome.

### Stable transformation of cacao with *AtFT* causes early flowering in somatic embryos

**Fig. 6:**
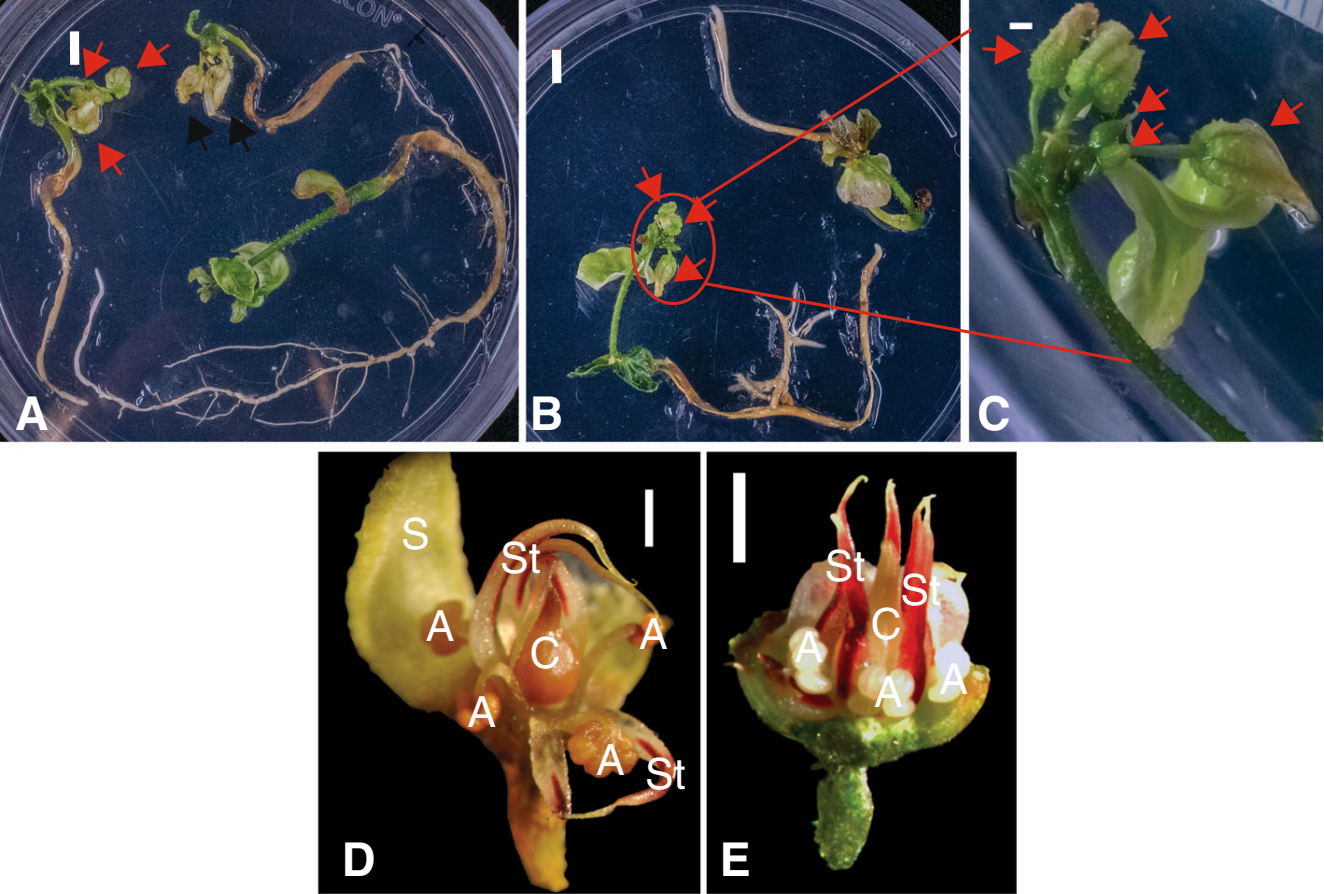
Early flowering of transgenic cacao overexpressing *AtFT.* **a** and **b** Floral buds (red arrows) and open flowers (black arrows) produced at the shoot apex of the shoots of E12-Ω::*AtFT* transgenic cacao in culture plates. **c** Close-up of (**b**) showing 5 total floral buds in a terminal cluster. **d** Dissected transgenic floral buds demonstrating morphologically complete flower. **e** ‘Control’ PSU-Sca6 flower from greenhouse grown tree for comparison. S = sepal, A = anther, St = staminode, C = fused carpels. Red scale bars = 5 mm. White scale bars = 1 mm

Having demonstrated the orthologous nature of *TcFT* and *AtFT* through phylogenetic, functional and gene expression analyses, we next transformed cotyledons from cacao secondary somatic embryos [50] with either *E-12 Ω::TcFT* or *E-12 Ω::AtFT* overexpression constructs. Transformations with both overexpression constructs resulted in regeneration of several abnormal embryos that were delayed in growth and had arrested growth without developing roots or shoots (data not shown). Only one transformation event with *E-12 Ω::AtFT* resulted in regeneration of five transgenic embryos that appeared normal during early development. The cotyledons of these embryos were excised and cultured in tissue culture to regenerate additional embryos and establish a transgenic line. To generate more embryos, regeneration was initiated from transgenic E-12 Ω*::AtFT* cotyledons multiple times. Approximately, 1 year after the original transformation, 15 transgenic embryos began to flower in tissue culture after the production of one or more true leaves. Single flowers or floral clusters were primarily produced at the shoot apex of transgenic plants (Fig. 6a-c), but flowers were occasionally observed to form in the axils of leaves (not shown). Shortly after floral production, transgenic embryos ceased growth and all shoot and root tissues died.

**Fig. 7:**
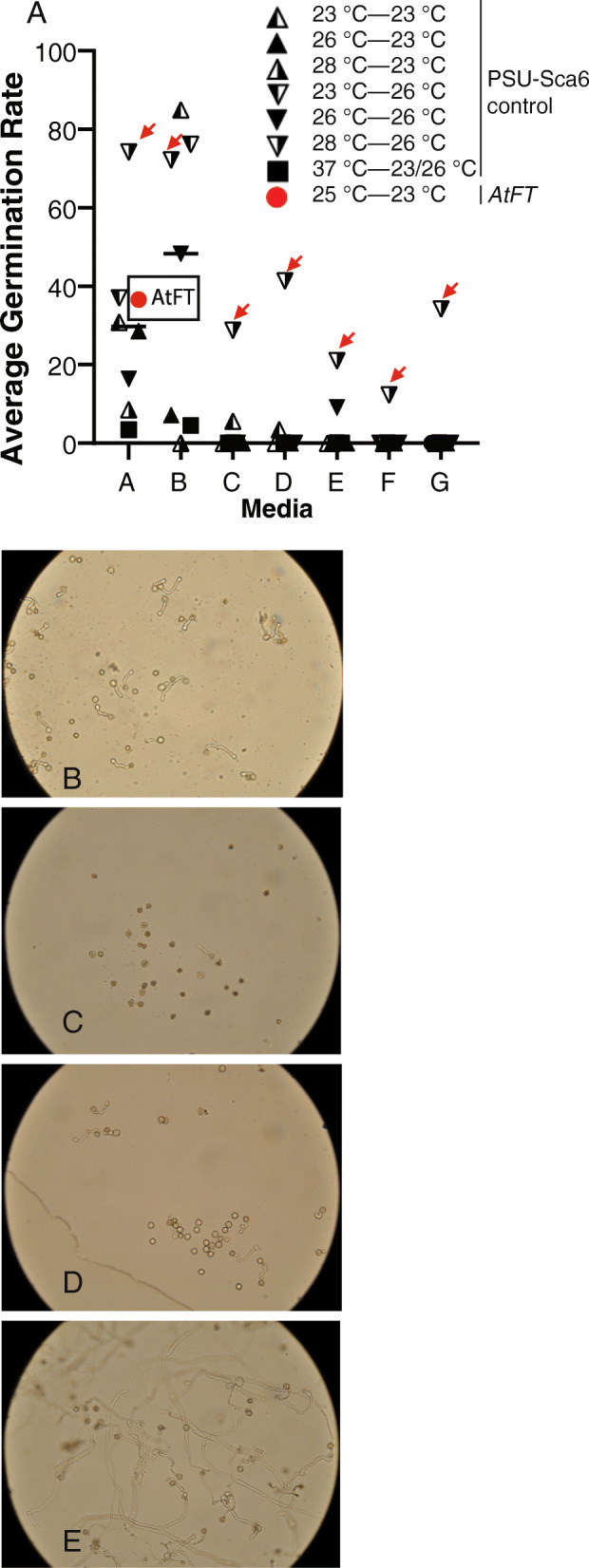
*AtFT* transgenic and PSU-Sca6 control cacao pollen viability assessed by in vitro germination. The germination rate for control PSU-Sca6 pollen was assayed at a range of experimental conditions. **a** and **e** Control pollen germinated optimally with flowers incubated at 28 °C pre-test and pollen tested at 26 °C. Red arrows in (**a**) highlight the consistency with which this experimental regime led to higher germination rates even in unfavorable media compositions. (A-D) *AtFT* pollen germinated at a similar average rate as pollen from control flowers assayed under similar conditions. **b**-**c** Micrographs of *AtFT* pollen in vitro germination; tested transgenic pollen displayed diverse germination rates as pictured. **d** Micrograph of control pollen in vitro germination in experimental conditions: media A, pre-test incubation of 23 °C, pollen assay at 23 °C. **e** Micrograph of control pollen germination at optimal experimental conditions. Pollen tubes in (**e**) are markedly longer than tubes in (**b**-**d**). Media compositions are listed in Table S4. °C—°C temperature in (**a**) legend indicate pre-test and pollen germination temperatures, respectively

Nine flowers produced by the tissue culture plants were dissected to assess morphological integrity (Fig. 6). All flowers observed contained the normal complement of floral organs, with 4 whorls as follows: an outer whorl having five sepals, a whorl of 5 petals, a whorl of 5 stamens and 5 staminodes, and a whorl containing 5 fused carpels. All *AtFT* transgenic flowers observed had reproductive structures (stamens, and carpels, of the innermost whorls) that were darker in appearance (brown vs. white) compared to the reproductive structures of flowers from of greenhouse grown PSU-Sca6 trees, the genotype used for the transformation (Fig. 6d-e). To determine if the precocious flowers were capable of producing viable pollen grains, the viability of pollen from *AtFT* transgenic flowers (*n* = 2) was evaluated alongside pollen from greenhouse grown PSU-Sca6 control flowers. Pollen from transgenic flowers, one tested at anthesis and one tested 1 day post-anthesis, exhibited greatly diverse germination rates (68.6 and 4.7%, respectively) with an average rate of 36.6%. This result is similar for PSU-Sca6 control flowers tested under similar experimental conditions: (Fig. 7a-d and Table S4). The highest germination rates for control pollen were recorded when flowers were incubated at 28 °C for 4 h pre-test and pollen was in vitro germinated at 26 °C (Table S4, Fig. 7a and e). Although these results demonstrated that the precocious flowers produced as a result of over-expression of *AtFT* in cacao somatic embryos produced viable pollen, we were unable to successfully pollinate flowers of greenhouse grown plants in several attempts (data not shown).

## Discussion

*FT* is a member of the CETS gene family*,* an ancient gene family with extant members found in all forms of life. In angiosperms, the complexity of this gene family varies widely. Close relatives to *T. cacao*, Arabidopsis and cotton comprise a relatively small family structure of six and eight members, respectively, while monocots *Zea mays* and wheat have expanded family structures of 23 and 19 *CETS* genes, respectively [10, 23, 31, 32]. In the present study, we identified seven highly conserved candidate family members of the *Theobroma cacao CETS* gene family, which is similar to the number of genes found in the closest relatives previously studied. Similar to cotton, cacao’s nearest living relative with a completed reference genome, cacao comprises just one functional florigen ortholog, while Arabidopsis contains two functional florigens (*AtFT* and *AtTSF*) [11–13, 51]. Furthermore, while the *TFL1/SP*-L clade has expanded in cotton to comprise five members, in cacao, this clade contains only three members, *TcTFL1, TcSP,* and *TcBFT*. Both cotton and cacao contain multiple *MFT*-L genes showing a duplication that could have occurred before the divergence of these species. In addition to the two shared *MFT* genes, cacao’s genome contains a third truncated *MFT*-L gene, *TcMFT-L3*, encoding a truncated small peptide comprised of the most critical residues necessary for *CETS* functionality.

In order to assess the role of *TcFT* in flowering time regulation, we overexpressed its coding sequence in late flowering *ft-10* Arabidopsis mutant where it restored flowering time and branching architecture to wild-type phenotype demonstrating *TcFT* to be a functional ortholog of *AtFT. FT* orthologs from numerous species overexpressed in Arabidopsis and crop species have resulted in early flowering.

In general, the expression of the *TcFT* is similar to the expression of *AtFT* [13, 38, 52]. Namely, the expression in both species is both developmentally and diurnally regulated. *FT* is a major integrator of several signal transduction pathways responsible for the induction of an angiosperm’s transition to reproductive growth [15, 53]. Comprehensive studies have shown that this role is conserved among many species, including photoperiodic and day-neutral plants. We find that *TcFT* expression increased with leaf maturity in a similar fashion to that of *AtFT* and well-studied tomato florigen, *SFT* [10]. This leaf expression pattern is consistent with *FT*’s role as a general accelerator of determinate growth or promoter to floral transitioning.

The *TcFT* gene was expressed in floral tissues, consistent with its demonstrated expression in Arabidopsis [54, 55]. As previously discussed, *AtFT* floral tissue expression was linked to stabilization of nearby inflorescence and floral meristems [38]. Cacao flowers initiate in axils of abscised leaves on the main branches and trunk of adult cacao trees. Inflorescences arise iteratively from the same spot on branches and eventually form floral cushion comprised of many compressed cincinnal cymes [56]. A survey of auxin concentrations in cacao cultivars having varied cushion density (number of flowers/cushion) showed a negative correlation between floral density and floral auxin concentrations [57]. In the same study, exogenous auxin application was positively linked to increased flower and fruit retention in incompatible pollinations leading the authors to conclude that hormonal levels control cacao self-incompatibility through a unspecified genetic factor. Our results demonstrating conservation of gene expression patterning with Arabidopsis *FT* suggests that *TcFT* might similarly stabilize cacao reproductive development by signaling nearby meristems to produce reproductive structures and that *TcFT* expression in floral tissues could impact cushion density. Additional studies conclusively linking *TcFT* floral expression changes in clones with contrasting cushion density phenotypes and/or endogenous auxin content could reveal an elusive link between *FT* and auxin in addition to discovering the genetic link to the hormonal control of cacao self-incompatibility.

Here we present the first report of FT-engineered early flowering in cacao. Our attempts to regenerate cacao embryos transformed with *TcFT* were unsuccessful with a limited number of transformed embryos dying off in early growth. It seems plausible that TcFT overexpression caused developmental abnormalities that did not allow normal embryos to successfully develop. It is possible that with weaker or more tissue specific promoters, we can overcome this obstacle. Interestingly, we were able to regenerate a single transgenic somatic embryo expressing *AtFT* (Fig. 6) that was used as an explant for establishment of a transgenic line via sequential somatic embryogenesis. Using established protocols, selected mature somatic embryos were transferred to conversion media for development into plantlets. Under conversion conditions in tissue culture, the plantlets developed one or more true leaves followed by development of flowers as a single flower or cluster of terminal flowers at the shoot apical meristem with normal morphology. It should be noted that for 20 years our research group has generated a large number of cacao transgenic somatic embryos using the Agrobacterium-mediated transformation method applied for this study, using the same binary vector containing various transgenes fused to E-12 Ω promoter and 35S terminator, and we have never observed flower development in tissue culture or early flower development in young somatic embryo-derived plantlets. However, our results are similar to the observed flowering in vitro of other plant species overexpressing *FT* orthologs. The first report of a juvenile transgenic tree producing inflorescences describes Agrobacterium-mediated transformation of male *Populus tremula x tremuloides* and female *P. tremula* stem with *35S::PtFT1* where floral development was observed 4 weeks post-transformation. The authors reported normal floral development, but noted that only weakly expressing lines were able to be regenerated in the greenhouse [58]. In apple, two reports described in vitro flowering using *35S::MdFT1* causing flowering of apple clones 8–12 month post transformation [59, 60]. Transgenic apple plants were also described to have a weak growth habit, often senescing and flowers occasionally showing abnormal morphologies [59].

In addition to normal floral morphology, pollen from *AtFT* transgenic plantlets was viable as demonstrated by the in vitro germination assay. This result suggests that transgenic pollen from cacao tissue culture has the potential to be used as donor genetic material in crossings that could accelerate cacao breeding dramatically. A drawback of the current protocol is the early death of the transgenic embryos after initial floral production. It is likely that constitutive *AtFT* expression in these embryos quickly drive all growing plant tissues to terminal states. In species, such as apple [61] and poplar [19] transgenic plant growth was improved by utilization of inducible promoters, such as heat-shock promoters. Likewise, constructs allowing for inducible/controlled expression of FT could be beneficial for transformation of cacao.

## Conclusions

We have identified and characterized members of the cacao *CETS* gene family and demonstrate that the candidate *TcFT* florigen gene is expressed in a tissue specific profile consistent with *FT* gene expression in other species. Overexpression of *TcFT* in a late-flowering Arabidopsis mutant partially restored normal wild-type flowering time demonstrating its potential for promoting the transition to flowering. Furthermore, heterologous expression of *AtFT* in cacao tissues resulted in the production of flowers in cacao somatic embryos, which produced viable pollen. Collectively our results support the conclusion that *TcFT* (Tc05v2_g009810) encodes an evolutionarily conserved functional ortholog of AtFT and that the mechanisms of floral induction control through FT are largely conserved between cacao and Arabidopsis.

## Methods

### Plant materials and growth conditions

Arabidopsis seeds were obtained from The Arabidopsis Biological Resource Center (Columbia-0 (Col-0) and *ft-10* (ABRC, stock # CS9869) and were germinated on soil or half-strength MS medium (PhytoTechnology Laboratories, Lenexa, KS, USA) supplemented with 1% sucrose. Seeds were stratified at 4 °C for 3 days and transferred to a Conviron walk-in chamber for growth with day lengths as indicated in the text (22/18 °C day/night) and light intensity of 120–150 μmol photons m^− 2^ s^− 1^ at leaf level. *Theobroma cacao* accessions Scavina-6 and a closely related accession PSU-Sca6, were propagated as rooted stem cuttings of greenhouse grown trees originally obtained from USDA ARS Subtropical Research Station in Mayaguez, Puerto Rico, and o. PSU-Sca6 trees used within these studies were trees originally obtained from USDA ARS Subtropical Research Station in Mayaguez, Puerto Rico and clonal propagated (by rooted stem cuttings) trees of these trees. Sca-6 and PSU-Sca6 trees were grown in pots in a silica sand and perlite mix (2:1) under greenhouse conditions. Importation and growth of these plants followed all relevant USDA guidelines and were grown in BL-2 level greenhouses regulated by the Penn State Office of Research Protections. Humidity was maintained at 60%, and the photoperiod was set to 16 h light/29 °C and 8 h dark/26 °C. Natural light was supplemented with 430-W high pressure sodium lamps as needed to maintain a minimum light level of 250 mmol m^− 2^ s^− 1^ PAR, while automatically retractable shading limited light levels to a maximum of 1000 mmol m^− 2^ s-^1^ PAR. Irrigation with one-tenth-strength Hoagland’s nutrient solution (160 ppm N) was applied daily at multiple times to maintain adequate moisture.

### Phylogenetic analyses

Cacao *CETS* genes were identified by BLASTp searches against two *Theobroma cacao* genomes: the Criollo B97–61/B2 v2 ([26, 27]; E-value cutoff 1E-10) and Matina1–6 v1.1 ([28, 29]; E-value cutoff 1E-05) genomes using Arabidopsis FT (AT1G65480.1), TFL1 (AT5G03840.1) and ATC (AT2G27550) protein sequences as queries [26, 28]. Functionally critical domains of predicted CETS polypeptide sequences from *T. cacao* were aligned with the corresponding domains of CETS proteins from Arabidopsis (*A. thaliana*), tomato (*Solanum lycopersicum*), cotton (*Gossypium hirsutum)*, and moss (*Physcomitrella patens*) using MUSCLE 3.8.425 implemented in Geneious Prime 2019.2.1 [62, 63]. A phylogenetic tree based on the multiple sequence alignment was constructed using the bootstrap test by the neighbor-joining method in Mega 7 [64, 65]. The optimal tree with the sum branch length = 5.57896991 is shown (Fig. 1). The percentage of replicate trees in which the associated taxa clustered together in the bootstrap test (1000 replicates) are indicated next to the branches [66]. The evolutionary distances were computed using the JTT matrix-based method and are in the units of the number of amino acid substitutions per site [67]. The analysis involved 43 amino acid sequences. All ambiguous positions were removed for each sequence pair. There was a total of 237 positions in the final dataset. The phylogenetic tree was rooted with MFT-L sequences from the distantly related moss *Physcomitrella patens*. Accession numbers for all protein sequences used in the analyses are listed in Supplementary Table 3 (Table S3).

### Vector construction

Cloning was by common molecular biology techniques [68]. Restriction endonucleases were from New England Biolabs (NEB, Ipswich, MA, USA). Oligonucleotides were synthesized by IDT (Coralville, IA, USA). All constructs were analyzed by restriction digest (NEB) and DNA sequence verification (Penn State Nucleic Acid Facility, University Park, PA, USA).

Total RNA was isolated from mature leaves of *T. cacao* Scavina-6 (100 mg) and from rosette leaves Arabidopsis Columbia-0 (100 mg), using Purelink Plant RNA Reagent (Life Technologies, Carlsbad, CA, USA) with minor alterations as follows: 1 mL of plant reagent was added to frozen ground tissue, 0.2 mL of 5 M NaCl was added to samples prior to chloroform extraction, 0.6 mL of chloroform was used in a first chloroform extraction, a second chloroform extraction was performed with equal volume of chloroform to aqueous layer, and all centrifugations were performed at 16,000 g. To obtain the coding sequences of *TcFT and AtFT,* 1 μg of total RNA from each plant species was treated with DNaseI (Thermo Fisher Scientific, Waltham, MA, USA) and reverse transcribed using an oligo dT_23_ primer and M-MLV RT (Promega, Madison, WI, USA). Corresponding fragments were PCR-amplified using Phusion polymerase (NEB) and primers TcFT_SpeI_f: 5′-CGA CTA GTA TGC CTA GAG AAA GAG ACC CCT TG-3′ and TcFT_HpaI_r: 5′-CGG TTA ACT CAT CGC CTC CGG CCT CC-3′ or AtFT_SpeI_f: 5′-CGA CTA GTA TGT CTA TAA ATA TAA GAG ACC-3′ and AtFT_HpaI_r: 5′-CGG TTA ACC TAA AGT CTT CCT CC-3′. PCR products were blunt cloned into cloning vector pMiniT2.0 (NEB) and transformed in chemically competent 10-beta *E. coli* cells according to manufactures instructions (give the kit and manufacture info here). Coding sequences from both species were released by SpeI/HpaI digestion and cloned into the same sites behind the E12-Ω promoter in binary vector pGZ12.0501 (GenBank: KF871320.1) to create E12-Ωpro::*TcFT vector* pGSp18.0102 (Fig. S1, GenBank MN856144) and E12-Ωpro::*AtFT* vector pGSp18.0129 (Fig. S2, GenBank MN856143).

### Arabidopsis transformations and phenotypic analysis

Binary vectors were introduced into Agrobacterium strain AGL1 by electroporation. The Arabidopsis *ft-10* mutant (ABRC stock # CS9869) was transformed with pGSh17.0404 (backbone vector control, GenBank MN856142), pGSp18.0102 (E12-Ωpro::*TcFT*), or pGSp18.0129 (E12-Ωpro::*AtFT*) via the floral dip method (Clough and Bent, 1998), and transformants were selected using kanamycin (100 mg l^− 1^). Transformed plants were analyzed in T1 generation. Post selection, T1 plants were transferred to soil and grown in 16/8 day/night conditions. Plants were phenotyped for time of flowering and architectural traits as previously described [69].

### Expression analyses

For spatiotemporal expression analysis, leaf tissue was harvested from 1.5 year-old (vegetative) and 2.5–3.5 year-old (flowering) Scavina-6 greenhouse grown plants between 11 am – 1 pm and flash frozen in liquid nitrogen. Three biological replicates of each tissue type were analyzed. Tissue was homogenized using mortar and pestle and total RNA was isolated using Purelink Plant RNA Reagent (Life Technologies) with minor modifications as described above. RNA samples were treated with DNase I (Thermo Fisher Scientific). 1.6 μg of RNA was used for cDNA synthesis using SuperScript IV Reverse Transcriptase (Thermo Fisher Scientific). To study the diurnal expression of *TcFT*, Scavina-6 mature (Stage E) leaf tissue was harvested from trees every four hours over a 24-h time course. Four biological replicates were harvested for each time point. Tissue was homogenized and RNA extracted as described above. 1.4 μg of RNA was used for cDNA synthesis using SuperScript IV Reverse Transcriptase (Invitrogen). All qRT-PCR reactions were performed using an ABI 7300 StepOnePlus Real-Time PCR system (Applied Biosystems, Foster City, CA) and SYBR Premix Ex Taq reagents (Takara Bio USA, Mountain View, CA) using the oligonucleotides indicated in Supplementary Table 5 (Table S5). Reactions were performed in 10 μL volumes with final primer concentrations of 0.4 μM. qPCR cycling parameters were: 95 °C for 10 min, 40 cycles of 95 °C for 15 s, 60 °C for 30 s, 72 °C for 40 s then dissociation curve analysis. Reactions were performed in technical triplicate. Quantitative RT-PCR data analysis including reference gene stability, ΔΔCt, and statistical analysis were conducted using qbase+ software, version 3.2 [70].

### Cacao stable transformation

In order to examine the functionality of *FT* within the cacao system, we transformed secondary PSU-Sca6 somatic embryo cotyledons as previously described [74] and with modification detailed below, separately, with *Agrobacterium tumefaciens* strain AGL1 containing one of vectors pGSh17.0404, pGSp18.0102, or pGSp18.0129. Transformation protocol modifications include: Bacterial cultures were grown at 28 °C overnight and optical density was measured for at 600 nm; 523 media (10 g/L sucrose, 8 g/L casein enzymatic hydrolysate, 4 g/L yeast extract, 2 g/L K_2_PO_4_, and 0.15 g/L MgSO_4_) was used for induction of the bacterial cultures; 30–35 cacao cotyledon explants were added to 50 mL Falcon containing agrobacterial cultures in 523 media; all sonication steps were performed for 100 s; explant infection was performed by shaking the Falcon tubes on their sides at 50 rpm and 28 °C for 20 min, followed by aspiration of bacterial culture before transferring the explants to solid tissue culture medium; co-cultivation of explants with *A. tumefaciens* on solid medium was performed for 72 h. Cultures were first observed at 4 weeks post culture initiation, followed by observations every other week as previously described [50]. The transgenic embryo expressing reporter gene eGFP was cultured and multiplied through de novo regeneration as previously described [50].

### Transgenic and control pollen in vitro germination

Flowers from transgenic embryos growing at 25 °C were excised immediately prior to the start of in vitro germination. Freshly-opened PSU-Sca6 (control) flowers from greenhouse trees grown (as described above) were harvested from 8 to 9 am and incubated in parafilm-sealed glass tissue culture jars for 4 h at one of four pre-incubation environments: room temperature (23 °C), 28 °C incubator, 37 °C incubator, or greenhouse (26 °C). Pollen from transgenic in vitro and control greenhouse flowers was germinated in vitro as previously described [75, 76] with modifications: 10 μL drops of liquid media was prepared onto glass micro slides. Three anthers were brushed onto the media drop to sow pollen. Test slides were incubated overnight sealed in moistened filter paper-lined 100 × 15 petri dishes. Transgenic pollen was evaluated only at 23 °C, while control pollen was evaluated at both 23 °C and in greenhouse conditions (26 °C) to determine optimal conditions. Media composition for evaluating pollen germination: 10% sucrose, 100 ppm boric acid, 300 ppm calcium nitrate, 200 ppm magnesium sulfate. Pollen from control flowers was also cultured on media with varied osmolytes: 20 or 30% sucrose and 0% or 15% PEG4000. Germination was determined by pollen tube expansion viewed at 20x magnification using a Reishart Microstar IV compound light microscope. Images were captured using Camera Control Pro 2 software (Nikon, USA) and a microscope-attached camera.

## Supplementary Information

**Additional file 1: Table S1.** BLASTp results using AtFT, AtTFL1 and ATC as queries against the *T. cacao* Belizian Criollo B97–61/B2 v2 genome.

**Additional file 2: Table S2.** BLASTp results using AtFT, AtTFL1 and ATC as queries against the *T. cacao* Matina1–6 v1.1 predicted proteome.

**Additional file 3: Table S3.** CETS proteins from moss (Physcomiterella patens), Arabidopsis, tomato (Solanum lycoperscium), and cotton (*Gossypium hirsutum*).

**Additional file 4: Table S4.** Average germination rate of transgenic and control cacao pollen in in vitro germination assay. Pollen from AtFT transgenic pollen was assay alongside pollen from PSU-Sca6 control flower in an in vitro germination assay. Control flowers were tested under varying assay conditions including varied osmolyte concentrations, pre-test and test incubation temperatures. AtFT pollen tested under low osmolyte and 23 °C test incubation showed an average germination of 36.6%.

**Additional file 5: Table S5.** Oligonucleotide primer sequenced used in gene expression RT-pPCR experiments.

**Additional file 6: Figure S1.** pGSp18.0102 vector map.

**Additional file 7: Figure S2.** pGSp18.0129 vector map.

## Data Availability

Data and materials are available by request to M. Guiltinan (mjg9@psu.edu) or for sequencing data, on NIH Genbank database.

## Abbreviations

ATC: Arabidopsis Centroradialis
BFT: Brother of FT and TFL1
CETS: Centroradialis/Terminal Flower1/Self Pruning
FT: Flowering Locus T
MFT: Mother of FT and TFL1
PEBP: Phosphytidylethanolamine Binding Proteins
SFT: Single Flower Truss
SP: Self Pruning
TFL1: Terminal Flower1

## References

[1] Ploetz R (2016). The Impact of Diseases on Cacao Production: A Global Overview. Cacao Diseases.

[2] Gutiérrez OA, Campbell AS, Phillips-Mora W (2016). Breeding for Disease Resistance in Cacao. Cacao Diseases.

[3] Motamayor JC, Risterucci AM, Lopez PA, Ortiz CF, Moreno A, Lanaud C (2002). Cacao domestication I: the origin of the cacao cultivated by the Mayas. Heredity (Edinb).

[4] Greathouse DC, Laetsch WM (1969). Structure and Development of the Dimorphic Branch System of *Theobroma cacao*. Am J Bot.

[5] Mustiga GM, Gezan SA, Phillips-Mora W, Arciniegas-Leal A, Mata-Quirós A, Motamayor JC (2018). Phenotypic Description of *Theobroma cacao* L. for Yield and Vigor Traits From 34 Hybrid Families in Costa Rica Based on the Genetic Basis of the Parental Population. Front Plant Sci.

[6] Swanson J, Carlson JE, Guiltinan MJ (2008). Comparative Flower Development in *Theobroma cacao* Based on Temporal Morphological Indicators. Int J Plant Sci.

[7] Zeevaart JAD (1976). Physiology of Flower Formation. Annu Rev Plant Physiol.

[8] Chailakhyan MK (1936). About the mechanism of the photoperiodic response. Dokl Akard Nauk SSSR.

[9] Vince-Prue D (1975). Photoperiodism in plants.

[10] Lifschitz E, Eviatar T, Rozman A, Shalit A, Goldshmidt A, Amsellem Z (2006). The tomato FT ortholog triggers systemic signals that regulate growth and flowering and substitute for diverse environmental stimuli. Proc Natl Acad Sci U S A.

[11] Sparks E, Wachsman G, Benfey PN (2013). Spatiotemporal signalling in plant development. Nat Rev Genet.

[12] Yang Y, Klejnot J, Yu X, Liu X, Lin C (2007). Florigen (II): It is a Mobile Protein. J Integr Plant Biol.

[13] Corbesier L, Vincent C, Jang S, Fornara F, Fan Q, Searle I (2007). FT protein movement contributes to long-distance signaling in floral induction of Arabidopsis. Science.

[14] Tamaki S, Matsuo S, Wong HL, Yokoi S, Shimamoto K (2007). Hd3a protein is a mobile flowering signal in rice. Science.

[15] Fornara F, de Montaigu A, Coupland G (2010). SnapShot: Control of flowering in Arabidopsis. Cell.

[16] Shalit-Kaneh A, Eviatar-Ribak T, Horev G, Suss N, Aloni R, Eshed Y (2019). The flowering hormone florigen accelerates secondary cell wall biogenesis to harmonize vascular maturation with reproductive development. Proc Natl Acad Sci U S A.

[17] McGarry RC, Klocko AL, Pang M, Strauss SH, Ayre BG (2017). Virus-Induced Flowering: An Application of Reproductive Biology to Benefit Plant Research and Breeding. Plant Physiol.

[18] Hoenicka H, Lehnhardt D, Polak O, Fladung M. Early flowering and genetic containment studies in transgenic poplar. iForest - Biogeosciences For. 2012;5(3):138. [cited 2020 Jan 3] Available from: https://iforest.sisef.org/contents/?id=ifor0621-005

[19] Hoenicka H, Lehnhardt D, Nilsson O, Hanelt D, Fladung M (2014). Successful crossings with early flowering transgenic poplar: interspecific crossings, but not transgenesis, promoted aberrant phenotypes in offspring. Plant Biotechnol J.

[20] Kotoda N, Wada M, Masuda T, Soejima J. The break -through in the reduction of juvenile phase in apple using transgenic approaches. Acta Hortic. 2003;(625):337–43. [cited 2017 Oct 27] Available from: http://www.actahort.org/books/625/625_40.htm

[21] Yamagishi N, Sasaki S, Yamagata K, Komori S, Nagase M, Wada M, et al. Promotion of flowering and reduction of a generation time in apple seedlings by ectopical expression of the *Arabidopsis thaliana* FT gene using the Apple latent spherical virus vector. Plant Mol Biol. 2011;75(1–2):193–204. [cited 2017 Oct 25] Available from: http://link.springer.com/10.1007/s11103-010-9718-0 10.1007/s11103-010-9718-0 21132560

[22] McGarry RC, Ayre BG (2012). Geminivirus-mediated delivery of florigen promotes determinate growth in aerial organs and uncouples flowering from photoperiod in cotton. PLoS One.

[23] Prewitt SF, Ayre BG, McGarry RC (2018). Cotton CENTRORADIALIS/TERMINAL FLOWER 1/SELF-PRUNING genes functionally diverged to differentially impact plant architecture. J Exp Bot.

[24] Kumar S, Stecher G, Suleski M, Hedges SB (2017). TimeTree: a resource for timelines, Timetrees, and divergence times. Mol Biol Evol.

[25] TimeTree: The Timescale of Life. [cited 2020 Nov 30]. Available from: http://www.timetree.org/

[26] Argout X, Salse J, Aury J-M, Guiltinan MJ, Droc G, Gouzy J (2011). The genome of *Theobroma cacao*. Nat Genet..

[27] Cocoa Genome Hub | Cocoa Genome Hub. [cited 2020 Nov 30]. Available from: https://cocoa-genome-hub.southgreen.fr/

[28] Motamayor JC, Mockaitis K, Schmutz J, Haiminen N, III DL, Cornejo O, et al. The genome sequence of the most widely cultivated cacao type and its use to identify candidate genes regulating pod color. Genome Biol. 2013;14(6):r53. [cited 2019 Jul 8] Available from: http://www.ncbi.nlm.nih.gov/pubmed/23731509. 10.1186/gb-2013-14-6-r53 PMC4053823 23731509

[29] Welcome to the Cacao Genome Project | Cacao Genome Database. [cited 2020 Nov 30]. Available from: https://www.cacaogenomedb.org/

[30] Shalit A, Rozman A, Goldshmidt A, Alvarez JP, Bowman JL, Eshed Y (2009). The flowering hormone florigen functions as a general systemic regulator of growth and termination. Proc Natl Acad Sci U S A.

[31] Danilevskaya ON, Meng X, Hou Z, Ananiev EV, Simmons CR (2008). A genomic and expression compendium of the expanded PEBP gene family from maize. Plant Physiol.

[32] McGarry RC, Prewitt SF, Culpepper S, Eshed Y, Lifschitz E, Ayre BG (2016). Monopodial and sympodial branching architecture in cotton is differentially regulated by the *Gossypium hirsutum* SINGLE FLOWER TRUSS and SELF-PRUNING orthologs. New Phytol.

[33] Wolabu TW, Zhang F, Niu L, Kalve S, Bhatnagar-Mathur P, Muszynski MG, et al. Three FLOWERING LOCUS T-like genes function as potential florigens and mediate photoperiod response in sorghum. New Phytol. 2016 [cited 2016 Apr 7]; Available from: http://www.ncbi.nlm.nih.gov/pubmed/26765652. 10.1111/nph.13834 26765652

[34] Ahn JH, Miller D, Winter VJ, Banfield MJ, Lee JH, Yoo SY, et al. A divergent external loop confers antagonistic activity on floral regulators FT and TFL1. EMBO J. 2006;25(3):605–614. [cited 2016 May 23] Available from: http://www.pubmedcentral.nih.gov/articlerender.fcgi?artid=1383534&tool=pmcentrez&rendertype=abstract 10.1038/sj.emboj.7600950 PMC1383534 16424903

[35] Goldshmidt A, Alvarez JP, Bowman JL, Eshed Y (2008). Signals Derived from *YABBY* Gene Activities in Organ Primordia Regulate Growth and Partitioning of *Arabidopsis* Shoot Apical Meristems. Plant Cell.

[36] Banfield M, Brady R (2000). The structure of Antirrhinum centroradialis protein (CEN) suggests a role as a kinase regulator. J Mol Biol.

[37] Mejía LC, Guiltinan MJ, Shi Z, Landherr L, Maximova SN (2012). Expression of Designed Antimicrobial Peptides in *Theobroma cacao* L. Trees Reduces Leaf Necrosis Caused by *Phytophthora* spp.

[38] Liu L, Farrona S, Klemme S, Turck FK (2014). Post-fertilization expression of FLOWERING LOCUS T suppresses reproductive reversion. Front Plant Sci.

[39] Laurie RE, Diwadkar P, Jaudal M, Zhang L, Hecht V, Wen J, et al. The Medicago FLOWERING LOCUS T homolog, MtFTa1, is a key regulator of flowering time. Plant Physiol. 2011;156(4):2207–2224. [cited 2015 Mar 4] Available from: http://www.plantphysiol.org/content/156/4/2207.short 10.1104/pp.111.180182 PMC3149922 21685176

[40] Kojima S (2002). Hd3a, a Rice Ortholog of the Arabidopsis FT Gene, Promotes Transition to Flowering Downstream of Hd1 under Short-Day Conditions. Plant Cell Physiol.

[41] Meng X, Muszynski MG, Danilevskaya ON. The FT-Like ZCN8 Gene Functions as a Floral Activator and Is Involved in Photoperiod Sensitivity in Maize. Plant Cell Online. 2011;23(3). [cited 2017 Jun 26] Available from: http://www.plantcell.org/content/23/3/942.long 10.1105/tpc.110.081406 PMC3082274 21441432

[42] Turck F, Fornara F, Coupland G (2008). Regulation and identity of florigen: FLOWERING LOCUS T moves center stage. Annu Rev Plant Biol.

[43] Lin M-K, Belanger H, Lee Y-J, Varkonyi-Gasic E, Taoka K-I, Miura E (2007). FLOWERING LOCUS T Protein May Act as the Long-Distance Florigenic Signal in the Cucurbits. Plant Cell Online.

[44] Yoo SK, Chung KS, Kim J, Lee JH, Hong SM, Yoo SJ (2005). CONSTANS activates SUPPRESSOR OF OVEREXPRESSION OF CONSTANS 1 through FLOWERING LOCUS T to promote flowering in Arabidopsis. Plant Physiol..

[45] Mitsuhara I, Ugaki M, Hirochika H, Ohshima M, Murakami T, Gotoh Y (1996). Efficient Promoter Cassettes for Enhanced Expression of Foreign Genes in Dicotyledonous and Monocotyledonous Plants. Plant Cell Physiol.

[46] Shi Z, Maximova S, Liu Y, Verica J, Guiltinan MJ (2013). The Salicylic Acid Receptor NPR3 Is a Negative Regulator of the Transcriptional Defense Response during Early Flower Development in Arabidopsis. Mol Plant.

[47] Shi Z, Maximova SN, Liu Y, Verica J, Guiltinan MJ (2010). Functional analysis of the *Theobroma cacao* NPR1 gene in arabidopsis. BMC Plant Biol.

[48] Liu Y, Shi Z, Maximova S, Payne MJ, Guiltinan MJ (2013). Proanthocyanidin synthesis in *Theobroma cacao*: genes encoding anthocyanidin synthase, anthocyanidin reductase, and leucoanthocyanidin reductase. BMC Plant Biol.

[49] Liu Y, Shi Z, Maximova SN, Payne MJ, Guiltinan MJ (2015). Tc-MYBPA is an Arabidopsis TT2-like transcription factor and functions in the regulation of proanthocyanidin synthesis in *Theobroma cacao*. BMC Plant Biol.

[50] Maximova SN, Alemanno L, Young A, Ferriere N, Traore A, Guiltinan MJ (2002). Efficiency, genotypic variability, and cellular origin of primary and secondary somatic embryogenesis of *Theobroma cacao* L. Vitr Cell Dev Biol - Plant.

[51] Yamaguchi A, Kobayashi Y, Goto K, Abe M, Araki T (2005). TWIN SISTER OF FT (TSF) acts as a floral pathway integrator redundantly with FT. Plant Cell Physiol.

[52] Liu L, Adrian J, Pankin A, Hu J, Dong X, von Korff M (2014). Induced and natural variation of promoter length modulates the photoperiodic response of FLOWERING LOCUS T. Nat Commun..

[53] Tränkner C, Lehmann S, Hoenicka H, Hanke MV, Fladung M, Lenhardt D, Dunemann F, Gau A, Schlangen K, Malnoy M, Flachowsky H (2010). Over-expression of an FT-homologous gene of apple induces early xowering in annual and perennial plants. Planta..

[54] Wenzel S, Flachowsky H, Hanke M-V (2013). The Fast-track breeding approach can be improved by heat-induced expression of the FLOWERING LOCUS T genes from poplar (*Populus trichocarpa*) in apple (Malus × domestica Borkh.). Plant Cell Tissue Organ Cult.

[55] Edgar RC. MUSCLE: a multiple sequence alignment method with reduced time and space complexity. BMC Bioinformatics. 2004;5(1):113 [cited 2019 Dec 3] Available from: http://www.ncbi.nlm.nih.gov/pubmed/15318951. 10.1186/1471-2105-5-113 PMC517706 15318951

[56] Geneious | Bioinformatics Software for Sequence Data Analysis. [cited 2020 Nov 30]. Available from: https://www.geneious.com/

[57] Kumar S, Stecher G, Tamura K. MEGA7: Molecular Evolutionary Genetics Analysis Version 7.0 for Bigger Datasets. Mol Biol Evol. 2016;33(7):1870–1874. [cited 2017 Jun 19] Available from: http://www.ncbi.nlm.nih.gov/pubmed/27004904. 10.1093/molbev/msw054 PMC8210823 27004904

[58] Schmid M, Davison TS, Henz SR, Pape UJ, Demar M, Vingron M, Schölkopf B, Weigel D, Lohmann JU (2005). A gene expression map of Arabidopsis thaliana development. Nat Genet.

[59] Bartolome R (1951). Cacao. Philipp J Agric.

[60] Hasenstein KH, Zavada MS (2001). Auxin modification of the incompatibility response in *Theobroma cacao*. Physiol Plant.

[61] Böhlenius H, Huang T, Charbonnel-Campaa L, Brunner AM, Jansson S, Strauss SH (2006). CO/FT regulatory module controls timing of flowering and seasonal growth cessation in trees. Science.

[62] Saitou N, Nei M. The neighbor-joining method: a new method for reconstructing phylogenetic trees. Mol Biol Evol. 1987;4(4):406–25. [cited 2017 Jun 19] Available from: http://www.ncbi.nlm.nih.gov/pubmed/3447015. 10.1093/oxfordjournals.molbev.a040454 3447015

[63] Felsenstein J (1985). Confidence Limits on Phylogenies: An Approach Using the Bootstrap. Evolution (N Y).

[64] Jones DT, Taylor WR, Thornton JM (1992). The rapid generation of mutation data matrices from protein sequences. Comput Appl Biosci.

[65] Sambrook J, Fritsch E, Maniatis T. Molecular cloning: a laboratory manual. 1989 [cited 2017 Aug 1]. Available from: https://www.cabdirect.org/cabdirect/abstract/19901616061

[66] Prewitt SF. Phylogenetic and Functional Characterization of Cotton (*Gossypium hirsutum*) CENTRORADIALIS/TERMINAL FLOWER1/SELF-PRUNING Genes. 2017 [cited 2019 Dec 4]; Available from: https://digital.library.unt.edu/ark:/67531/metadc1062895/

[67] Qbase: Innovative data services and solutions | Qbase: Innovative data services and solutions. [cited 2020 Nov 30]. Available from: https://qbase.com/

[68] Maximova S, Miller C, Antúnez de Mayolo G, Pishak S, Young A, Guiltinan MJ (2003). Stable transformation of *Theobroma cacao* L. and influence of matrix attachment regions on GFP expression. Plant Cell Rep.

[69] Aneja M, Gianfagna T, Ng E, Badilla I (1992). Carbon Dioxide and Temperature Influence Pollen Germination and Fruit Set in Cocoa. Am Soc Hortic Sci.

[70] Brewbaker JL, Kwack BH (1963). The essential role of calcium ion in pollen germination and pollen tube growth. Am J Bot.

