# Supplementary material for: Inter-species functional compatibility of the Theobroma cacao and Arabidopsis FT orthologs: 90 million years of functional conservation of meristem identity genes
Source: BMC Plant Biol. 2021 May 14;21:218. doi: 10.1186/s12870-021-02982-y (PMC8122565; doi:10.1186/s12870-021-02982-y)
Supplement: Supplementary file 1 — Additional file 1: Table S1. BLASTp results using AtFT, AtTFL1 and ATC as queries against the T. cacao Belizian Criollo B97–61/B2 v2 genome. [file 12870_2021_2982_MOESM1_ESM.pdf]

Supplemental Table 1: BLASTp results using AtFT, AtTFL1 and ATC as queries against the *T. cacao* Belizian Criollo B97-61/B2 v2 genome.

Locus ID	Designation	Protein Size	AtFT result		AtTFL1 result		ATC result	
			E-value	% Identity	E-value	% Identity	E-value	% Identity
Tc05v2_g009810	TcFT	174 aa	1E-100	76.4	1E-68	55.7	1E-65	55.9
Tc05v2_g007510	TcTFL1	172 aa	4E-67	56.3	3E-90	71.1	1E-90	70.9
Tc09v2_g023800	TcSP	174 aa	3E-65	55.1	6E-93	71.2	3E-104	80
Tc03v2_g014270	TcBFT	173 aa	2E-66	55.4	3E-81	63.2	2E-84	66.3
Tc03v2_g003780	TcMFT-L1	173 aa	1E-54	49.1	1E-54	47.4	1E-53	50.3
Tc06v2_g016620	TcMFT-L2	176 aa	2E-49	45.2	1E-57	50.3	1E-51	49.7
Tc06v2_g016640	TcMFT-L3	149 aa	5E-16	29.1	1E-17	32.9	1E-18	33.6
