# Supplementary material for: Inter-species functional compatibility of the Theobroma cacao and Arabidopsis FT orthologs: 90 million years of functional conservation of meristem identity genes
Source: BMC Plant Biol. 2021 May 14;21:218. doi: 10.1186/s12870-021-02982-y (PMC8122565; doi:10.1186/s12870-021-02982-y)
Supplement: Supplementary file 2 — Additional file 2: Table S2. BLASTp results using AtFT, AtTFL1 and ATC as queries against the T. cacao Matina1–6 v1.1 predicted proteome. [file 12870_2021_2982_MOESM2_ESM.pdf]

Supplementary Table 2: BLASTp results using AtFT, AtTFL1 and ATC as queries against the *T. cacao* Matina1-6 v1.1 predicted proteome.

| | | | AtFT result | | AtTFL1 result | | ATC result | |
|---|---|---|---|---|---|---|---|---|
| Locus ID | Designation | Protein Size | E-value | % Identity | E-value | % Identity | E-value | % Identity |
| Thecc1EG023287 | TcFT | 174 aa | 4E-80 | 76.4 | 5E-53 | 57.1 | 7E-52 | 55.9 |
| Thecc1EG022560 | TcTFL1 | 172 aa | 8E-53 | 56.3 | 9E-71 | 71.1 | 2E-70 | 70.9 |
| Thecc1EG041439 | TcSP | 174 aa | 4E-51 | 55.1 | 3E-73 | 71.3 | 1E-80 | 80 |
| Thecc1EG015117 | TcBFT | 173 aa | 2E-48 | 51.3 | 6E-59 | 58.6 | 1E-61 | 61.5 |
| Thecc1EG012687 | TcMFT-L1 | 173 aa | 4E-43 | 44.9 | 2E-38 | 42.9 | 2E-41 | 47.4 |
| Thecc1EG030010 | TcMFT-L2 | 176 aa | 3E-38 | 44.9 | 5E-45 | 50.3 | 2E-40 | 50.3 |
