# Supplementary material for: Inter-species functional compatibility of the Theobroma cacao and Arabidopsis FT orthologs: 90 million years of functional conservation of meristem identity genes
Source: BMC Plant Biol. 2021 May 14;21:218. doi: 10.1186/s12870-021-02982-y (PMC8122565; doi:10.1186/s12870-021-02982-y)
Supplement: Supplementary file 3 — Additional file 3: Table S3. CETS proteins from moss (Physcomiterella patens), Arabidopsis, tomato (Solanum lycoperscium), and cotton (Gossypium hirsutum). [file 12870_2021_2982_MOESM3_ESM.pdf]

Supplementary Table 3: CETS proteins from moss (*Physcomiterella patens*), Arabidopsis, tomato (*Solanum lycoperscium*), and cotton (*Gossypium hirsutum*).

Protein	Species	Accession
PpMFT1	*Physcomiterella patens*	ACN54543.1
PpMFT2	*Physcomiterella patens*	ACN54544.1
PpMFT3	*Physcomiterella patens*	ACN54546.1
PpMFT4	*Physcomiterella patens*	ACN54547.1
AtFT	*Arabidopsis thaliana*	AAF03936.1
AtTSF	*Arabidopsis thaliana*	BAA77840.1
AtTFL1	*Arabidopsis thaliana*	AED90661.1
AtATC	*Arabidopsis thaliana*	NP_180324.1
AtBFT	*Arabidopsis thaliana*	Q9FIT4.1
AtMFT	*Arabidopsis thaliana*	Q9XFK7.1
SlSP2G	*Solanum lycoperscium*	AAO31791.1
SlSP3D	*Solanum lycoperscium*	AAO31792.1
SlSP5G	*Solanum lycoperscium*	AAO31793.1
SlSP9D	*Solanum lycoperscium*	AAO31795.1
SlSP	*Solanum lycoperscium*	NP_001233974.1
SlBFT-L1	*Solanum lycoperscium*	Solyc01g009560
SlBFT-L2	*Solanum lycoperscium*	Solyc01g009580
SlBFT-L3	*Solanum lycoperscium*	Solyc03g026050
SlMFT	*Solanum lycoperscium*	Solyc03g119100
SlSP11D	*Solanum lycoperscium*	Solyc11g008660
GhSFT-A_t	*Gossypium hirsutum*	Gh_A08G2015
GhSFT-D_t	*Gossypium hirsutum*	Gh_D08G2407
GhBFT-L1-A_t	*Gossypium hirsutum*	Gh_Sca15601
GhBFT-L1-D_t	*Gossypium hirsutum*	Gh_D08G1087
GhBFT-L2-A_t	*Gossypium hirsutum*	Gh_A11G0088
GhBFT-L2-D_t	*Gossypium hirsutum*	Gh_D11G0092
GhSP-A_t	*Gossypium hirsutum*	Gh_A07G0997
GhSP-D_t	*Gossypium hirsutum*	Gh_D07G1075
GhTFL-L1-A_t	*Gossypium hirsutum*	Gh_A09G2442
GhTFL-L1-D_t	*Gossypium hirsutum*	Gh_D09G1320
GhTFL-L2-A_t	*Gossypium hirsutum*	Gh_A04G0520
GhTFL-L2-D_t	*Gossypium hirsutum*	Gh_D04G0971
GhMFT-L1-A_t	*Gossypium hirsutum*	Gh_A09G2391
GhMFT-L1-D_t	*Gossypium hirsutum*	Gh_D09G1658
GhMFT-L2-A_t	*Gossypium hirsutum*	Gh_Sca004734G03
GhMFT-L2-D_t	*Gossypium hirsutum*	Gh_D05G1586
