# Supplementary material for: Inter-species functional compatibility of the Theobroma cacao and Arabidopsis FT orthologs: 90 million years of functional conservation of meristem identity genes
Source: BMC Plant Biol. 2021 May 14;21:218. doi: 10.1186/s12870-021-02982-y (PMC8122565; doi:10.1186/s12870-021-02982-y)
Supplement: Supplementary file 5 — Additional file 5: Table S5. Oligonucleotide primer sequenced used in gene expression RT-pPCR experiments. [file 12870_2021_2982_MOESM5_ESM.pdf]

Supplementary Table 5: Oligonucleotide primer sequenced used in gene expression RT-pPCR experiments.

Primer Name	Primer Sequence 5' --> 3'	Gene Target ID
TcFT nt 229 F	AGT GAC CCA AAC CTG AGA GA	
TcFT nt 315 R	CAC AAC TTC TTG CCC AAA GC	Tc05v2_g009810
TcTUB1 RT F	GGA GGA GTC TCT ATA AGC TTG CAG TTG G	
TcTUB1 RT R	ACA TAA GCA TAG CCA GCT AGA GCC AG	Tc06v2_g000280
TcSUMO nt 384 F	TCC TGA ACA AAC TGA AGG CT	
TcSUMO nt477 R	CAG AGG TGG GTA CTG CTT GG	Tc01v2_g005340
TcCULLIN nt 2024 F	GAC GGT ATG CCA TTG ATG CCT	
TcCULLIN nt 2140 R	AAT CAG GCT TGA ACA TGC GG	Tc04v2_g023180
