# Supplementary material for: Inter-species functional compatibility of the Theobroma cacao and Arabidopsis FT orthologs: 90 million years of functional conservation of meristem identity genes
Source: BMC Plant Biol. 2021 May 14;21:218. doi: 10.1186/s12870-021-02982-y (PMC8122565; doi:10.1186/s12870-021-02982-y)
Supplement: Supplementary file 6 — Additional file 6: Figure S1. pGSp18.0102 vector map. [file 12870_2021_2982_MOESM6_ESM.pdf]

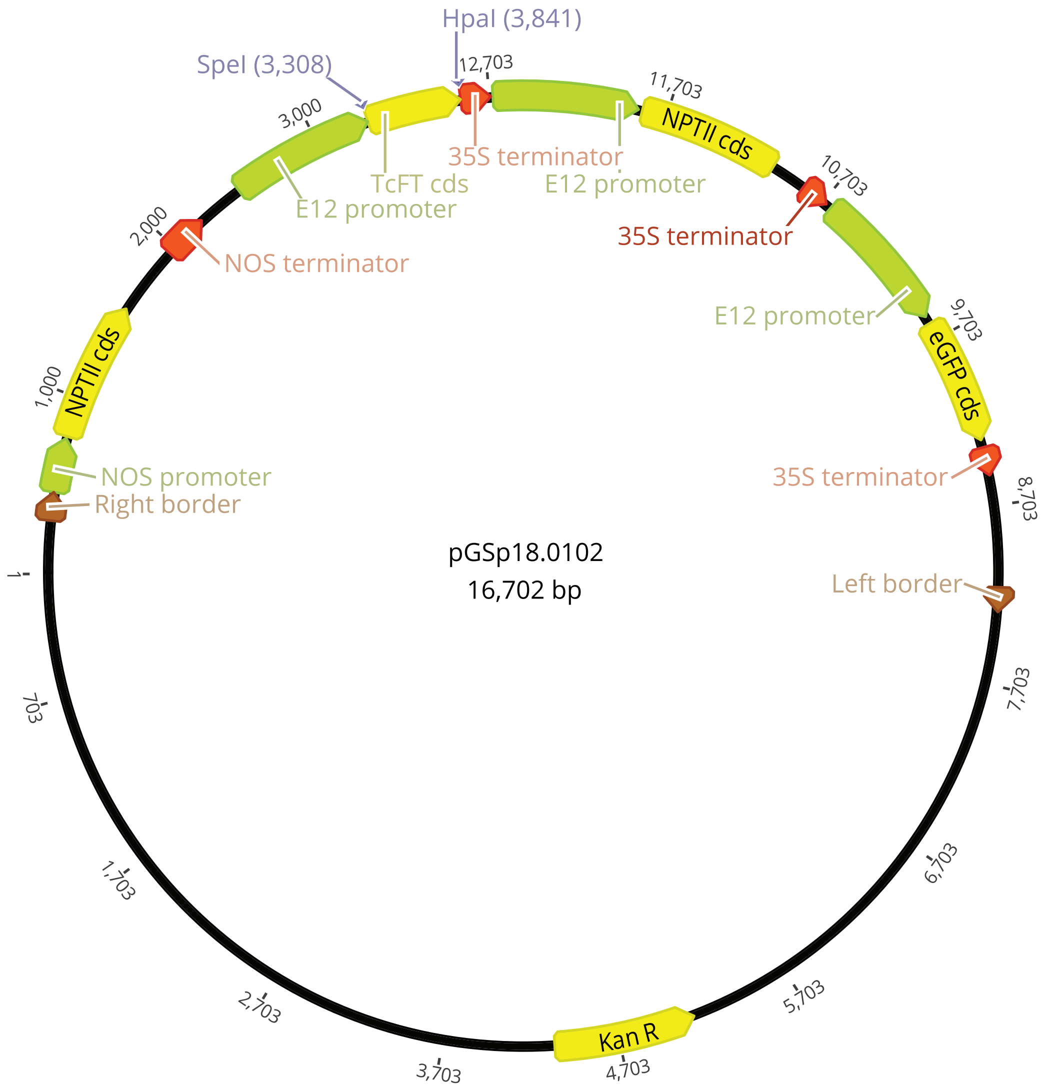

Figure S1: pGSp18.0102 vector map. Illustration of *TcFT* overexpression vector used to transform *T. cacao* PSU-Sca6 explants and Arabidopsis *ft-10* mutant in reported studies. The coding sequence of *TcFT* from Scavina6 genotype was cloned into entry vector pMiniT2.0 then subcloned into the SpeI and HpaI sites of pGZ12.0501 (Genbank KF871320.1). *TcFT* expression in the binary construct is driven by the constitutive E12 promoter.
