# Supplementary material for: Inter-species functional compatibility of the Theobroma cacao and Arabidopsis FT orthologs: 90 million years of functional conservation of meristem identity genes
Source: BMC Plant Biol. 2021 May 14;21:218. doi: 10.1186/s12870-021-02982-y (PMC8122565; doi:10.1186/s12870-021-02982-y)
Supplement: Supplementary file 7 — Additional file 7: Figure S2. pGSp18.0129 vector map. [file 12870_2021_2982_MOESM7_ESM.pdf]

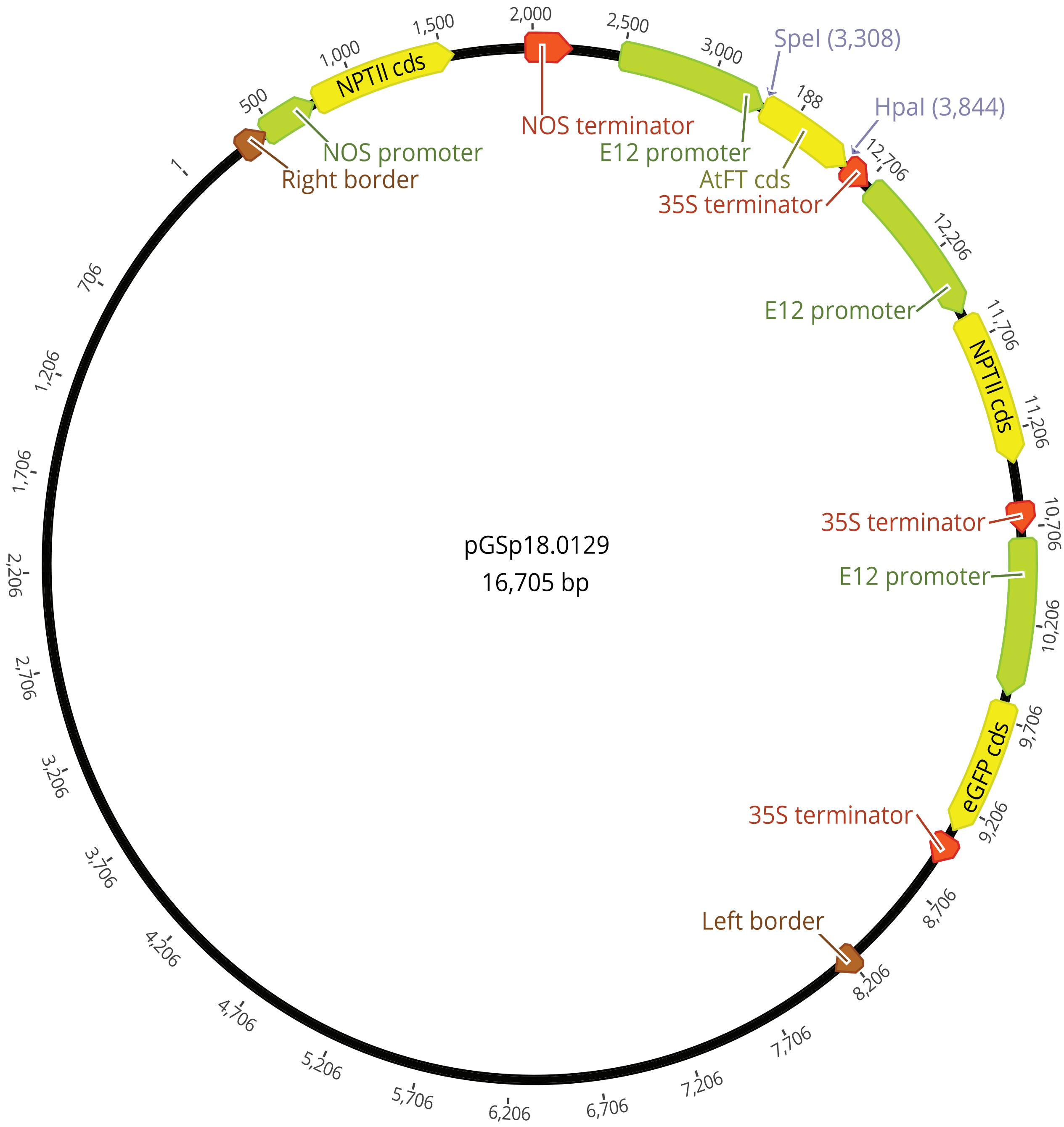

Figure S2: pGSp18.0129 vector map. Illustration of *AtFT* overexpression vector used to transform *T. cacao* PSU-Sca6 explants and Arabidopsis *ft-10* mutant in reported studies. The coding sequence of *AtFT* from Arabidopsis Columbia CS70,000 was cloned into entry vector pMiniT2.0 then subcloned into the SpeI and HpaI sites of pGZ12.0501 (Genbank KF871320.1). *AtFT* expression in the binary construct is driven by the constitutive E12 promoter.
