# Supplementary material for: Inter-species functional compatibility of the Theobroma cacao and Arabidopsis FT orthologs: 90 million years of functional conservation of meristem identity genes
Source: BMC Plant Biol. 2021 May 14;21:218. doi: 10.1186/s12870-021-02982-y (PMC8122565; doi:10.1186/s12870-021-02982-y)
Supplement: Supplementary file 4 — Additional file 4: Table S4. Average germination rate of transgenic and control cacao pollen in in vitro germination assay. Pollen from AtFT transgenic pollen was assay alongside pollen from PSU-Sca6 control flower in an in vitro germination assay. Control flowers were tested under varying assay conditions including varied osmolyte concentrations, pre-test and test incubation temperatures. AtFT pollen tested under low osmolyte and 23 °C test incubation showed an average germination of 36.6%. [file 12870_2021_2982_MOESM4_ESM.pdf]

Supplemental Table 4. Average germination rate of transgenic and control cacao pollen in *in vitro* germination assay. Pollen from *AtFT* transgenic pollen was assay alongside pollen from PSU-Sca6 control flower in an *in vitro* germination assay. Control flowers were tested under varying assay conditions including varied osmolyte concentrations, pre-test and test incubation temperatures. *AtFT* pollen tested under low osmolyte and 23 °C test incubation showed an average germination of 36.6%.

**Media Sucrose w/v % →**			**10**									**20**						**30**					
**Media PEG4000 w/v %**			**0**						**15**			**0**			**15**			**0**			**15**		
**Media plant extract**			**n/a**			**ICS-1 pistil**			**n/a**														
**Media Designation**			**A**			**B**			**C**			**D**			**E**			**F**			**G**		
	**Pre-assay**	**Assay**	**Average germination rate and Standard Error**																				
**Flower Type**	**Incubation Temperature**		AVG	SE	N	AVG	SE	N	AVG	SE	N	AVG	SE	N	AVG	SE	N	AVG	SE	N	AVG	SE	N
PSU-Sca6 control	23 °C	23 °C	30.9	19.3	2	84.9	0.0	1	5.7	0.0	1	3.5	0.0	1	0.0	0.0	1	0.0	0.0	1	0.0	0.0	1
	26 °C		28.7	11.6	2	7.2	0.0	1	0.0	0.0	1	0.0	0.0	1	0.0	0.0	1	0.0	0.0	1	0.0	0.0	1
	28 °C		8.6	6.0	2	0.0	0.0	1	0.0	0.0	1	0.0	0.0	1	0.0	0.0	1	0.0	0.0	1	0.0	0.0	1
	23 °C	26 °C	37.1	26.2	2	76.1	0.0	1	0.0	0.0	1	0.0	0.0	1	0.0	0.0	1	0.0	0.0	1	0.0	0.0	1
	26 °C		16.4	11.6	2	48.3	0.0	1	0.0	0.0	1	0.0	0.0	1	9.1	0.0	1	0.0	0.0	1	0.0	0.0	1
	28 °C		74.2	3.4	2	72.2	0.0	1	28.8	0.0	1	41.4	0.0	1	21.1	0.0	1	12.3	0.0	1	34.2	0.0	1
	37 °C	23/26 °C	3.5	2.5	2	4.5	3.2	2	0.0	0.0	2	0.0	0.0	2	0.0	0.0	2	0.0	0.0	2	0.0	0.0	2
AtFT		23 °C	36.6	22.6	2	n/a																	
